# TMC-SNPdb: an Indian germline variant database derived from whole exome sequences

**DOI:** 10.1093/database/baw104

**Published:** 2016-07-09

**Authors:** Pawan Upadhyay, Nilesh Gardi, Sanket Desai, Bikram Sahoo, Ankita Singh, Trupti Togar, Prajish Iyer, Ratnam Prasad, Pratik Chandrani, Sudeep Gupta, Amit Dutt

**Affiliations:** ^1^Integrated Genomics Laboratory, Advanced Centre for Treatment Research Education in Cancer (ACTREC); ^2^Department of Medical Oncology, Tata Memorial Centre, Mumbai, Maharashtra 410012, India

## Abstract

Cancer is predominantly a somatic disease. A mutant allele present in a cancer cell genome is considered somatic when it’s absent in the paired normal genome along with public SNP databases. The current build of dbSNP, the most comprehensive public SNP database, however inadequately represents several non-European Caucasian populations, posing a limitation in cancer genomic analyses of data from these populations. We present the **T**ata **M**emorial **C**entre-SNP **d**ata**b**ase (TMC-SNPdb), as the first open source, flexible, upgradable, and freely available SNP database (accessible through dbSNP build 149 and ANNOVAR)—representing 114 309 unique germline variants—generated from whole exome data of 62 normal samples derived from cancer patients of Indian origin. The TMC-SNPdb is presented with a companion subtraction tool that can be executed with command line option or using an easy-to-use graphical user interface with the ability to deplete additional Indian population specific SNPs over and above dbSNP and 1000 Genomes databases. Using an institutional generated whole exome data set of 132 samples of Indian origin, we demonstrate that TMC-SNPdb could deplete 42, 33 and 28% false positive somatic events post dbSNP depletion in Indian origin tongue, gallbladder, and cervical cancer samples, respectively. Beyond cancer somatic analyses, we anticipate utility of the TMC-SNPdb in several Mendelian germline diseases. In addition to dbSNP build 149 and ANNOVAR, the TMC-SNPdb along with the subtraction tool is available for download in the public domain at the following:

**Database URL:** http://www.actrec.gov.in/pi-webpages/AmitDutt/TMCSNP/TMCSNPdp.html

## Introduction

Somatic mutations sequentially accumulate in cancer cell genomes. In addition, a typical cancer genome contains several polymorphic ‘normal’ germline variants (1–3). Subtracting the tumor DNA variants against matched normal DNA derived from the same individual and those polymorphic in the population is, therefore, essential to identify an exclusive somatic event (4). Apropos, a critical aspect of any tumor genome sequence analysis involves depletion of paired normal variants followed by depletion of residual variants from public databases of common single nucleotide polymorphism (SNP) such as dbSNP (5) and 1000 Genomes Project (6). A sequence variant not observed in matched normal derived genome sequence and absent from public SNP database is considered somatic in origin. Adopting such an analytical approach ensures filtering of paired-germline and population-specific polymorphic variants from dbSNP and 1000 Genomes Project for Caucasian population (7).

However, despite depletion against dbSNP, unknown SNPs especially those with lower minor allele frequency not represented in dbSNP, are likely to confound somatic mutation analyses in studies involving non- Caucasian and non-European Caucasian populations (5). Two exhaustive initiatives addressing this issue are the publicly available exome variation datasets: NHLBI Exome Sequencing Project (https://esp.gs.washington.edu/EVS/) and Exome Aggregation Consortium (ExAC) (http://exac.broadinstitute.org/) (8). Information gathered from these studies is an integral part of variant annotation tools like Annovar (9).

Multiple studies such as the Indian Genome Variation Consortium (10, 11) and HUGO Pan- Asian SNP Consortium (12) have described the genomic distinctiveness of Indian population based on varying allele frequency of known SNPs, complex origin, genetic diversity (13–16), and high variation of male lineages (Y-chromosome) within the population (17, 18). However, a concerted effort to comprehensively identify and catalogue novel SNPs present exclusively in Indian population is yet to be undertaken. Lack of Indian specific SNP database has been an important impediment in cancer research, especially in efforts to discover bona fide novel somatic mutations.

Here, we describe **T**ata **M**emorial **C**entre-SNP **d**ata**b**ase ‘TMC-SNPdb’ as the first, open source, freely available database of unique germline variants obtained from whole exome data of 62 ‘normal’ samples from tongue, gallbladder, and cervical cancer patients of Indian origin. ‘TMC-SNPdb’ is presented with an easy-to-use graphic user interface feature to enable researchers to call true somatic mutations by depleting against Indian population specific SNPs, in addition to those already catalogued in dbSNP and 1000 Genomes databases. We demonstrate that ‘TMC-SNPdb’ effectively filters false positive somatic events across 75 tumor whole exome data.

## Materials and methods

### Ethical approval and informed consent

The sample set and study protocol was approved by Institutional Review Board (project no. 116 for cervical adenocarcinoma samples; project no. 88 for head and neck cancer samples, project 104 for gallbladder cancer samples). Cervical squamous carcinoma whole exome data have been described earlier in (19). Written informed consent was obtained from all patients.

### Extraction of DNA

All ‘normal’ tissue samples under study were verified by an onco-pathologist to not harbor any cancer. A total of 62 samples ‘normal’ samples (16 peripheral venous blood and 46 adjacent normal tissue) were obtained for analysis: peripheral venous blood from patients with cervical squamous cell carcinoma (*n* = 10), cervical adenocarcinoma (*n* = 18) (adjacent normal tissue; *n* = 12 and peripheral venous blood; *n* = 6) and adjacent normal tissue from patient with tongue squamous cell carcinoma (*n* = 23) and gallbladder (*n* = 11) were obtained from Tata Memorial Hospital (TMH). Genomic DNA from tissues was extracted using DNeasy blood and tissue DNA extraction kit (Qiagen) according to manufacturer’s instructions. Quantification of DNA was assessed using Nanodrop 2000c Spectrophotometer (Thermo Fisher Scientific Inc.) and DNA integrity was determined by resolving on 0.8% Agarose gel. DNA was also quantified using Qubit ds DNA BR assay kit (Life Technologies, USA). DNA samples showing DNA concentration >50 ng/µl and intact DNA visualized on agarose gel were used for whole exome sequencing.

### Exome capture, library preparation and sequencing

Three different library preparation kits were used to prepare libraries for different tumor types (Supplementary Table S1). First, TruSeq Exome Enrichment kit (v2 and v3, Illumina) was used to capture 62 Mb region (>3 40 000 probes) of human genome comprising 201 121 exons representing 20 974 gene sequences, including 5′UTR, 3′UTR, microRNAs and other non-coding RNA. For exome library preparation, two microgram genomic DNA was sheared using Covaris (Covaris Inc) for generating fragment sizes of 200–300 bp. Fragments end repairing, purification, A-tailing, adaptor ligation and quality control steps were carried out using TruSeq DNA Sample Prep Kit (Illumina) following manufacturer’s instructions. Qualitative and quantitative analysis of genomic DNA libraries were performed using High Sensitivity DNA chip on 2100 Bioanalyzer (Agilent) and qPCR with KAPA Library Quant Kit (Kapa Biosystems). Exome enrichment was done by incubation at 93 °C for 1 min (decreasing 2 °C per cycle for 18 cycles) followed by 58 °C for 19 h in ABI 9700 PCR system (Life Technologies) using 500 ng of genomic libraries.

Second, NimbleGen SeqCap EZ Exome Library (v3.0, Roche) targeting 64 Mb of the human genome was also used for library preparation. The protocol was adopted from the manufacture’s application note (http://www.nimblegen.com/products/lit/NimbleGen_SeqCap_EZ_SR_Pre-Captured_Multiplexing.pdf). Sequencing libraries were exome captured and later quality-controlled using a bioanalyzer (Agilent 2100) and libraries were qPCR quantified using KAPA Library Quant Kit (Kapa Biosystems) prior to cluster generation on an Illumina cBOT.

Third, SureSelect Human All Exon Kit, v5 (Agilent Technologies, Santa Clara, CA, USA) was also used to capture 50 Mb of the human genome using > 5 50 000 probes. One microgram of genomic DNA was utilized for library preparation and a similar protocol was followed as previously stated. Eluted exome-enriched library fragments were PCR amplified and purified.

qPCR quantified 7 pmol of 6-plex DNA library pool was loaded per lane on flow cell (Flow Cell v3) to generate clusters using TruSeq PE Cluster Kit v3-cBot-HS kit and clustered flow was sequenced for 201 and 301 cycles on HiSeq-1500 and NextSeq System (Illumina) using TruSeq SBS Kit v3 (Illumina), respectively.

### Exome sequencing variant analysis for TMC-SNP database

Paired-end raw sequence reads were mapped to human reference genome (build hg19) using BWA v. 0.6.2 (20). Quality control analysis of bam files was carried out using qualimap (v0.7.1) (21). Mapped reads were then used to identify and remove PCR duplicates using Picard tools v.1.74 (http:broadinstitute.github.io/picard/). Base quality score recalibration and indel re-alignment were performed and variants were called from each sample separately using GATK Unified Genotyper (version 2.5-2) (22).

### Development of TMC-SNP database

To restrict our analysis to high quality germline variants we applied filters of minimal base coverage and recurrence in cohort. In house developed scripts (Awk and Perl) were used to merge all 62 VCF files from normal tissues and mutational recurrence was calculated. We applied a standard filter of coverage ≥5 reads for altered alleles. Additionally, we included variants with coverage ≤5 but recurrent in ≥4 normal samples. Using these filters, we identified high quality variants in the dataset. High quality variants were further annotated using COSMICdb (version 68) (23) and dbSNP (version 142) (5). Remaining variants were further depleted against dbSNP and COSMICdb to remove all known somatic and germline variants. Finally, all remaining variants constitute the TMC-SNP database. A detailed schema of resource and data representation is provided in Supplementary Figure 3.

### Application of TMC-SNP database in analyzing tumor samples

GATK (version 2.5-2) and MuTect (version 1.0.2) (24) were utilized to generate raw variants of tumor samples and filtered against its matched normal . Variants obtained from GATK and MuTect were merged and variants having ≥5 reads for altered allele were kept for further downstream analysis. Similar analysis was carried out for three cancer types. Comparison with dbSNP(version142) and COSMICdb(version 68) was performed using in-house developed scripts in Perl and Awk which were later used to calculate the percentage changes in variants in different cancer type post filtration with dbSNP and TMC-SNP database. Functional annotation of variants was performed using Oncotator (variant annotation tool) (25).

### Germline variant subtraction program

TMC-SNPdb is distributed as a SQLite file containing variant information table. A companion tool for subtraction of germline variants from tumor sample has been developed in python (version 3.4). It depends on PyVCF (version ≥ 1.6) and sqlite3 python packages. The variants in TMC-SNPdb are characterized by a unique combination of chromosome number, genomic position, reference allele, altered allele for each variant and subtraction was carried out based on these unique fields for each variant in VCF file. The tool is an executable compatible with Linux operating system and has been tested on several Linux platform such Red Hat (version 6.5), Fedora (version 22) and Ubuntu (version 14.04). It can be executed using a command line interface (‘TMC-SNP’) or a graphical user interface (GUI) (‘TMC-SNP_GUI’). The GUI mode additionally depends on TKinter python library (version ≥ 2.4). Moreover, the tool has a feature which lets users create their own germline variant database from VCF format files of normal samples. The output obtained from the tool is in VCF format. Detailed user manual with snapshots of the GUI and schematic representation of overall usages are provided in Supplementary file 1 and Supplementary Figure S2.

### Availability of supporting data

The raw sequence data has been deposited at the ArrayExpress (http://www.ebi.ac.uk/arrayexpress/experiments/E-MTAB-4618), hosted by the European Bioinformatics Institute (EBI). The ‘TMC-SNPdb’ has been submitted to Annovar (http://annovar.openbioinformatics.org/en/latest/user-guide/download/) and dbSNP (http://www.ncbi.nlm.nih.gov/SNP/snp_viewTable.cgi?handle=TMC_SNPDB) for public access.

## Results

### Development of TMC-SNP database

**Figure 1.**
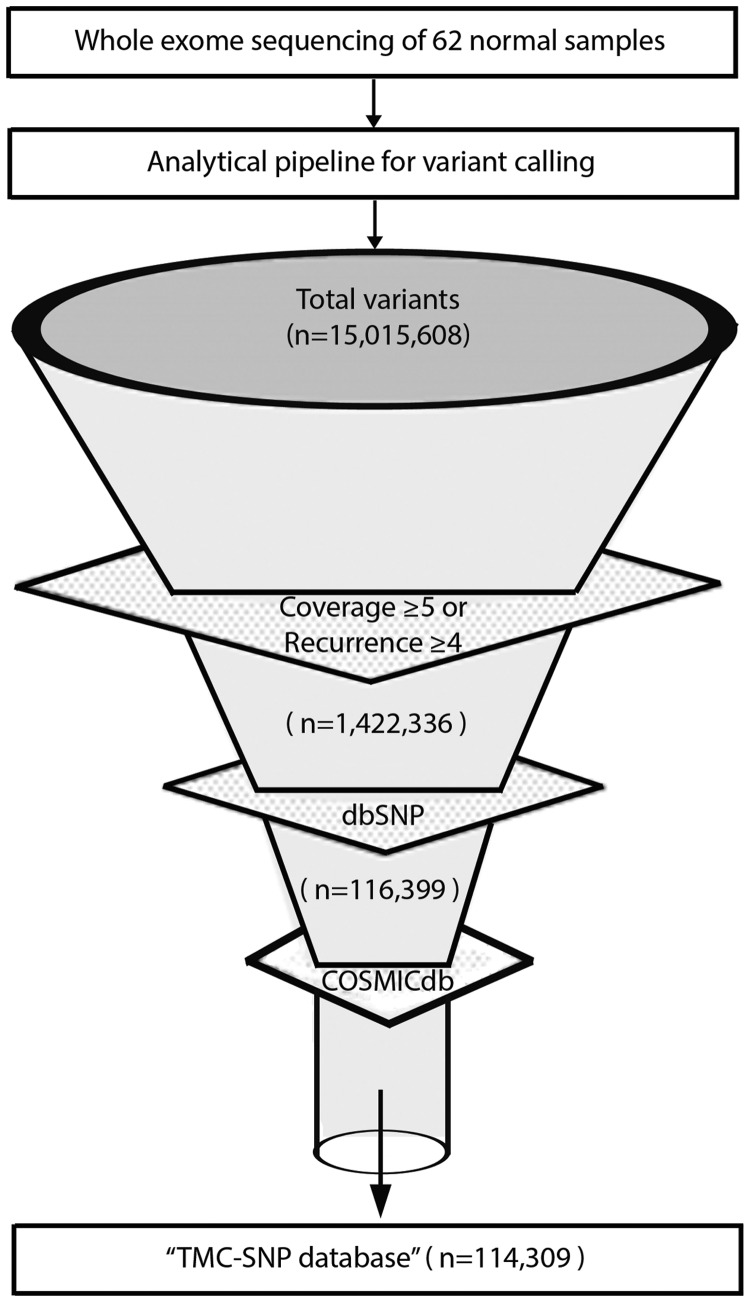
Development of TMC-SNPdb using whole exome sequencing. Schematic flow representation of steps followed during development of TMC-SNP database. The whole exome sequencing of 62 normal tissue obtained from three different tissues of cancer patients was performed and analysed using GATK (Genome Analysis Tool Kit) to generate VCF files. Raw variants obtained were further filtered using mentioned criteria to find a list of variants absent in dbSNP v142 and COSMICdb v68. Remaining variants constitutes the ‘TMC-SNPdb’ shown at the end of the funnel.

We analyzed whole exome sequencing at a median of 88x coverage for 62 normal samples derived from cancer patients, comparable with similar reports (26) as detailed in Supplementary Table S1. Of note, coverage among 4 of 62 samples were <30× due to high duplication reads and low yield in these samples. Germline mutations were called using GATK (22): a total of 15 015 608 germline variants were identified across the complete dataset. As shown in Figure 1, standard quality filters of minimal 5× coverage or recurrence in at least four samples for each variant led to about 90% reduction in raw variants (see Materials and Methods section for details). The remaining 1 422 336 variants of higher confidence were further depleted against dbSNP v142. 1 305 937 of 1 422 336 variants, constituting 92% SNPs were depleted. To remove variants known to be somatically associated with cancer in literature but figured as a germline event in our study (most likely due to inadequate or non-uniform coverage of their paired normal samples), we further depleted 2090 variants (2%) overlapping with COSMICdb with an assumption of these variants to be false somatic events in our data set. Finally, a total of 114 309 variants were identified after filtering with dbSNP and COSMICdb as a pool of previously unknown germline variants of high confidence recurring in the Indian population to constitute the ‘TMC-SNPdb’.

**Table 1:** Application of TMC-SNPdb across cancer types to filter germline variants in Indian population

S.No.	Cancer type	Total variants	Number of samples	Number and percentage (along row) of novel variants		Overall reduction by TMC-SNPdb post dbSNP depletion
				Post dbSNP depletion	Post TMC-SNPdb depletion	
1	Tongue cancer	613 055	24	84 001 (13.7%)	48 182 (7.8%)	42.6%
2	Cervical cancer	923 547	34	99 032 (10.7%)	71 594 (7.7%)	27.7%
3	Gall-bladder	328 245	17	26 530 (8%)	17 682 (5.3%)	33.3%

Total number of variants observed for each cancer types and reduction in number and percent variants post dbSNP and post TMC-SNPdb subtraction is tabulated for three cancer types. Number of samples analysed across tumor is also denoted.

### Characteristic features of TMC-SNP database

**Figure 2.**
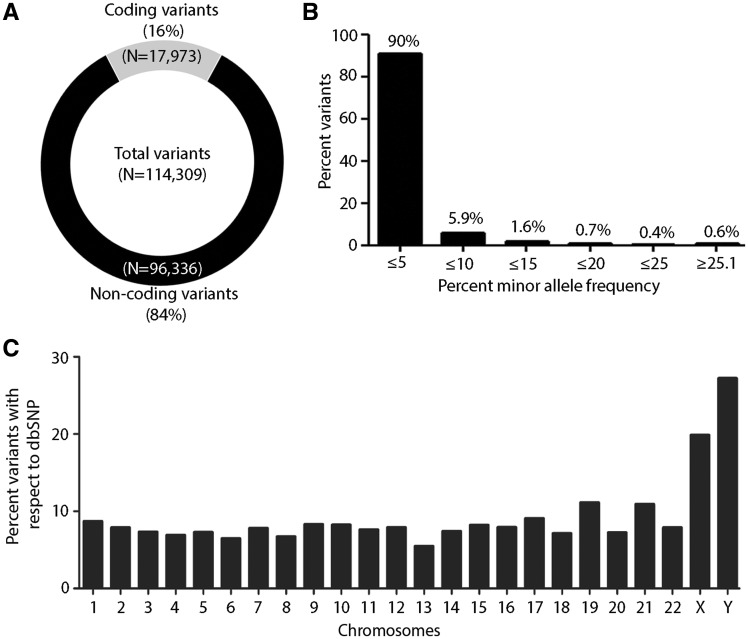
Overall overview of characteristic features of TMC-SNP database. **(A)** Circle plot of coding and non-coding variants obtained in the dataset. **(B)** Percent minor allele frequency distribution of variants in ‘TMC-SNPdb’ across 62 normal samples. Percentage frequencies are presented on the top of each bar. **(C)** Genome-wide distribution of percent frequency of variants obtained in each chromosome as compared with dbSNP database.

A total 114 309 variants were annotated using Oncotator for functional features (25). A distribution pattern of coding (∼17 973) and non-coding variants germline variants (∼96 336) is shown in Figure 2A. Of 17 973 coding variants, 11 466 were of non-synonymous (NS) (∼63%) and 6507 were synonymous variants (S) (∼36%) with NS/S ratio 1.76, consistent with previous reports for exome data from normal samples (27, 28). Furthermore, we observed a high frequency of missense (∼58%) and silent variants (∼30%) as compared with indel (∼3%), nonsense (∼2%) and splice site (∼6%) region (Supplementary Figure 1A). Of all the SNPs present in TMC-SNPdb, distribution varied across the genome as follows: protein-coding exon (15.7%), intron (40%), IGR (25.8%), 3′UTR (9.5%), 5′UTR (2.37%), RNA (3.74%) and lincRNA (1.7%), consistent with earlier report from exome sequencing data (Supplementary Figure S1B) (29, 30). Next, we computed the allele frequency of all 114 309 variants present in the TMC-SNPdb, across 62 samples. Given that TMC-SNPdb predominantly enlists low frequency germline variants prevalent among Indian population, similar to 1000 genomes and ExAC wherein about 99% of SNPs are estimated to have a minor allele frequency over 1% (8, 26), Similarly, in TMC-SNPdb >90% of variants present exist at a minor allele frequency≤5% (Figure 2B).

Furthermore, a comparative measure of variability added by the TMC-SNPdb variants to the known pool of SNPs per chromosome was reckoned following comparison with dbSNP variants across the genome. Interestingly, we found maximal variability at the Y-chromosome among 2418 of 8885 SNPs (27%), while the distribution of the variants across the autosomal chromosomes was found to be uniformly distributed among 106 184 of 1 346 256 SNPs (7.6%) similar to the dbSNP (Figure 2C). Of note, variants at Y-chromosome tend to be more localized geographically than those of mitochondrial DNA (mtDNA) and autosomes, which is reflective of the degree of inter-population genetic differences (31–33). Y-chromosomes have been shown to harbor population specific unique haplotype in Indian population and have frequently been used as a marker for studying human demographic history (34, 35). The higher variability at the Y-chromosome found in TMC-SNPdb is thus consistent with several earlier reports describing a high variation of male lineages within Indian population (17, 18) that further emphasizes the Indian specific characteristics of the TMC-SNPdb germline variants, and a need for distinct Indian specific germline database .

Finally, a significant characteristic feature of TMC-SNPdb is the companion subtraction tool with command line and GUI based interface. The user can deplete their data set against TMC-SNPdb or create a customized normal variant database. The program has been tested to run on various Linux platforms such Fedora, Ubuntu and Red Hat operating systems. (Detailed user manual and snapshot of different steps have been provided in Supplementary Materials S1, S2 and Supplementary Figure S2). Using the companion tool on an 8GB machine, it takes 56 and 72 min to filter standard VCFs containing 115 884 and 227 779 raw variants (provided as example file with tool) against the TMC-SNPdb variants, respectively.

### Application of ‘TMC-SNPdb’ in depleting germline variants predominant among indian population

With the flexibility of using GUI interface or through the command line (refer to Supplementary Material S1), we tested the robustness and practical utility of ‘TMC-SNPdb’ across various cancer types to infer the extent of depletion of population specific variants over and above the dbSNP. We analyzed 132 samples of three cancer types: head and neck cancer (*n* = 43), cervical cancer (*n* = 62) and gallbladder cancer (*n* = 27). Significant fold reduction of variants was observed following TMC-SNPdb subtraction in addition to depletion by dbSNP in all cancer types studied. Of 613 055 variants found across 24 head and neck cancer tumor samples about 92% SNPs were depleted post dbSNP subtraction with 84 001 candidate somatic variants. Subsequent depletion using TMC-SNPdb identified 35 819 additional variants as Indian specific germline variants existing at varying frequency in normal Indian population. In overall, TMC-SNPdb allowed us to filter an additional 42.6% of post dbSNPs depleted SNPs. in 24 tongue cancer samples (Table 1). Similarly, TMC-SNPdb significantly reduced about 33.3% and 27.7% SNPs in 17 gallbladder and 34 cervical tumor whole exome data, as tabulated in detail in Table 1.

## Discussion

TMC-SNPdb is a freely available open access Indian population specific germline variant database consisting of 114 309 germline variants using whole exome sequencing of 62 normal tissues from patients with different types of cancer. Its usage is analogous to depletion against pooled normal variants from unrelated normal samples of Indian origin for paired or orphan tumor samples. The utility of subtraction against pooled normal variants has been well described as a reference for depletion, especially for orphan tumor samples wherein paired normal variant data for the tumor samples are not available (36–39). Our dataset and companion tool can be used, along with other public databases, as ‘normal’ counterpart to identify disease specific somatic mutations, especially in cancer exome studies. Using TMC-SNPdb across 132 whole exome data of 3 tumor types, we show that it can significantly deplete false positive somatic variants.

TMC-SNPdb is presented with a companion program with command line or user-friendly GUI interface for non-computational biologists. It has two built-in features: first, a user can input tumor VCF to subtract against TMC-SNPdb and second, create a custom database of germ line mutation with the availability of multiple normal VCF files and then subtract with tumor VCF to deplete germ line variants. The subtraction program has been tested on several Linux platforms such as Fedora, Ubuntu and Red Hat system. Because it is an open source tool, it could be further modified to alter filtering parameters for analysis indicative of its expandability and universal applicability on Linux platforms.

There are two major limitations of TMC-SNPdb database. First, it is presumed that a sample derived from cancer patients represents ‘normal’ genome variation. However, because of their diseased status, a fraction of such individuals are likely to harbor cancer predisposing variants in their germline. Any such germline variant that is novel in Indian population (not yet included in Caucasian databases) and which predisposes to cancer (e.g. in *BRCA 1* gene) would be characterized as ‘normal’ population variation in TMC-SNPdb. Thus, this database will be limited in application to analyses that seek to evaluate germline predisposition to cancer. Second, majority of ‘normal’ samples were obtained from sites adjacent to a tumor with histopathological based inspection for the absence of tumor cells. However, it is possible that these tissues harbors some bona fide somatic mutations due to effect of field cancerization (40, 41). Thus, depleting against TMC-SNPdb could potentially ‘over-subtract’ mutations that are bona fide somatic. To minimize this possibility, we have filtered TMC-SNPdb variants against COSMIC database to remove any known cancer related somatic variants. However, there remains a residual potential for missing ‘somatic’ mutations that are novel in tumors of Indian patients and present in adjacent ‘normal’ tissue. With these caveats, we believe that TMC-SNPdb with its companion tool is a step towards fulfilling a significant unmet need for an Indian population ‘normal’ variant database, especially in somatic mutation analyses in tumors from Indian patients.

In summary, TMC-SNPdb is an open source database of ‘normal’ germline variants derived from Indian—non-European Caucasian—population, not yet included in the public databases with predominant Caucasian representations. It comes along with a companion tool that can apply this information for somatic cancer genome analyses by depleting against the TMC-SNPdb. This database is flexible to accommodate the need for customization by allowing inclusion of similar datasets from additional individuals.

## Supplementary data

Supplementary data are available at *Database* Online.

Supplementary Data
