# Supplementary material for: TMC-SNPdb: an Indian germline variant database derived from whole exome sequences
Source: Database (Oxford). 2016 Jul 9;2016:baw104. doi: 10.1093/database/baw104 (PMC4940432; doi:10.1093/database/baw104)
Supplement: Supplementary Data [file supp_2016_baw104_index.html]

TMC-SNPdb: an Indian germline variant database derived from whole exome sequences — Supplementary Data 

# TMC-SNPdb: an Indian germline variant database derived from whole exome sequences

## Supplementary Data

files

- Supplementary Data - zip file
