# Supplementary material for: TMC-SNPdb: an Indian germline variant database derived from whole exome sequences
Source: Database (Oxford). 2016 Jul 9;2016:baw104. doi: 10.1093/database/baw104 (PMC4940432; doi:10.1093/database/baw104)

**TMC-SNPdb –Variant subtraction tool user manual**

**Dependencies:**

TMC-SNPdb subtraction tool depends on three python libraries sqlite3, Tkinter (for GUI version) and pyvcf (version >= 0.6.7). These libraries can be installed using following commands:

**Get and Install sqlite**

**sqlite3** (http://www.sqlite.org/download.html)

wget http://www.sqlite.org/sqlite-autoconf-3070603.tar.gz

tar xvfz sqlite-autoconf-3071502.tar.gz

cd sqlite-autoconf-3071502

./configure --prefix=/usr/local

make

sudo make install

**Get and Install PyVcf**

**pyvcf** (http://pyvcf.readthedocs.org/en/latest/)

wget https://pypi.python.org/packages/source/P/PyVCF/PyVCF-0.6.7.tar.gz

tar xvfz PyVCF-0.6.7.tar.gz

sudo python setup.py install

**Get and Install Python TKinter**

python TKinter library for executing the GUI mode

sudo apt-get install python-tk

**Installation:**

To install 'tmc-snpdb' on your Linux system, please follow step 1 & 2.

**1)** Untar TMC-SNPdb package using following command:

> tar xvf tmcsnpdb1.0.tar.gz

**2)** Run the INSTALL script (as administrator) on your system

> chmod +x INSTALL

> sudo sh INSTALL

**3)** Subtraction tool runs in two modes, Graphical user interface (GUI) and command line (CMD). Use 'tmc-snpdb' command on the terminal to execute it in CMD mode; Use 'tmc-snpdb-gui' to run the subtraction tool in GUI mode.

**1) Running Subtraction program in Command line mode:**

**For CMD mode use the following command:**

$ tmc-snpdb

Usage: tmc-snpdb [options]

**Options:**

**-h**, --help show this help message and exit

**-i** Input tumor VCF file (required)

**-o** Output file after TMC-SNPdb subtraction (Optional)

**-l** Load a custom normal variations database (with tmc-snpdb schema - refer README/ SCHEMA document in the package). Give SQLITE file as input (Optional)

**--vcf_dir** Directory path containing VCF files containing germline variants. Required for creating custom database.

**--sql** Output germline variant database. Required for creating custom database.

**Example:**

*use the help option

tmc-snpdb -h

or

tmc-snpdb --help

**Subtraction of user tumor vcf against TMC-SNPdb.**

*To subtract germline variants from tumor VCF using TMC-SNPdb, use the command (without specifying output file name).

tmc-snpdb –i input.vcf –o output.vcf (Optional)

**Create a custom germline database:**

User can create their own germline database with a set of normal/germline variant VCF files. The following command can be used;

$ tmc-snpdb --vcf-dir directory/path/to/vcf --sql output.sqlite

The output SQLite file is created with the following schema:

**chr -** holds chromosome number data, e.g. 'chr2'

**pos -** integer storing the position on the chromosome, e.g. 190023

**orig** – reference base as found in the reference genome, e.g. 'A'

**change -** altered base found in the samples, e.g. 'T'

**reccur -** recurrence of a particular change (A->T as in the above example) in across samples

**Schema to create a custom database**

Following is sql syntax could be used to create custom sqlite database file format

CREATE TABLE tmcsnpdb

( "chr" TEXT,

"pos" INTEGER,

"orig" TEXT,

"change" TEXT,

"reccur" INTEGER

);

Output from this program can be loaded for subtraction from tumor samples using the '-l' option of the program.

**For example:**

$ tmc-snpdb -i tumor.vcf -l custom_database.sql

**Test Run:**

Test VCF files are provided in the "data/test_vcf" directory. This directory contains two files; test1.vcf (227779 variants) and test2.vcf (115884 variants)

**Command line mode:**

To subtract germline variants from tumor VCF using TMC-SNPdb, use the command

$ tmc-snpdb –i data/test_vcf/test1.vcf –o test1_output.vcf

'-o' - output is an optional argument

Output file after subtraction from test1_output.vcf and test2_output.vcf will contains 224330 and

114388 variants, respectively.

**In GUI mode:**

Click on "UPLOAD A VCF FILE", select a file in /data/test_vcf

Directory in the file dialogue box. Then click on "RUN" to subtract variants.

On an Intel-i5-3210M CPU @2.5GHz x 4-32 bit Ubuntu (14.04) system with 8 GB RAM takes 72 minutes to process test1.vcf and 56 minutes to process test2.vcf.

**2) Running Subtraction program in Graphical User Interface (GUI) mode:**

**TMC-SNPdb GUI main window**

(1)

(2)


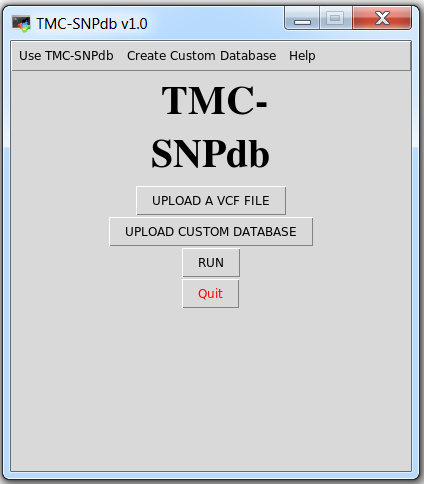


(3)

(6)

(5)

(4)

**Options 1-6 denoted by arrow are as described below**

1. Click to open menu and upload VCF file for germline subtraction against TMC-SNPdb.
2. Click to open menu and upload VCF directory from which a germline database is to be build.
3. Click to upload VCF file for germline subtraction against TMC-SNPdb.
4. Click to upload a custom database for germline subtraction.
5. Run the subtraction program.
6. Quit TMC-SNPdb.

**Menu to upload VCF file Select VCF file to upload**


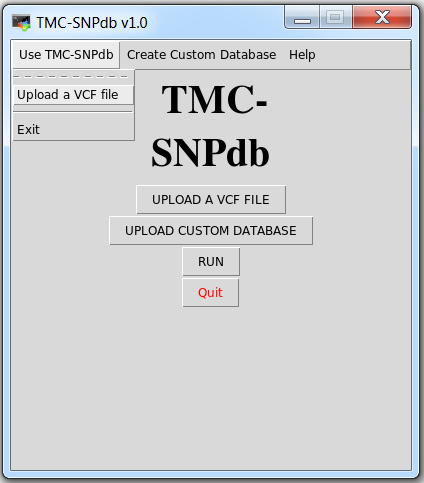

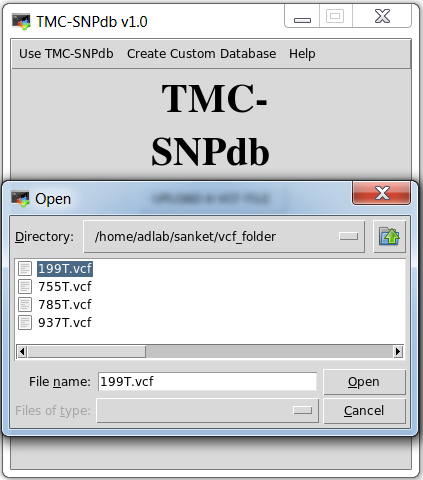


**Select a directory for creating custom germline database**

**Menu to upload VCF directory**


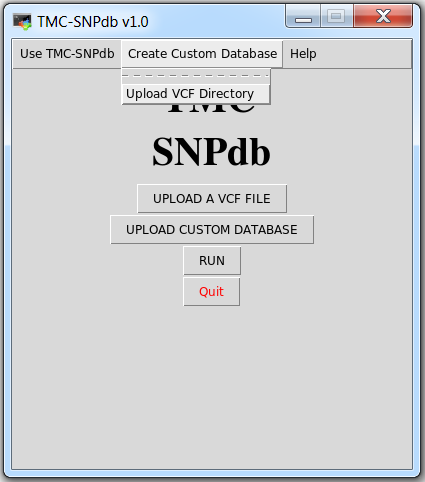

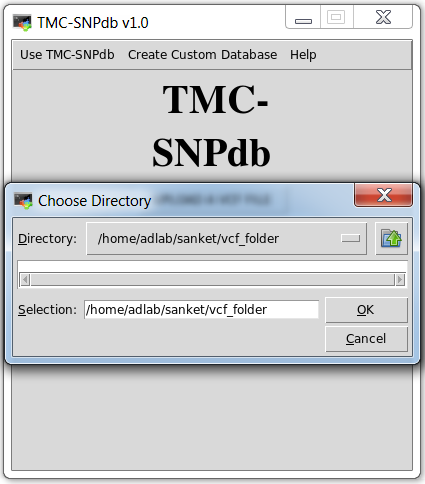


**Error message if vcf files not selected**

**Confirm to create database**


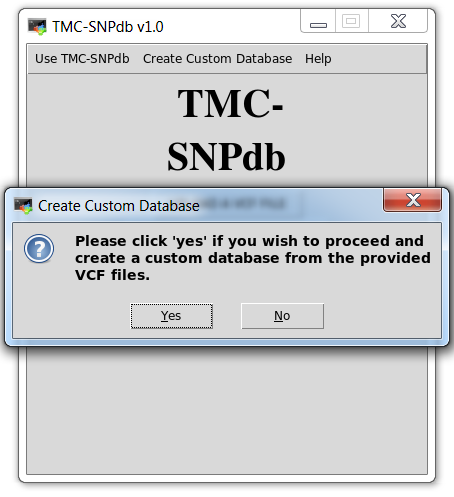

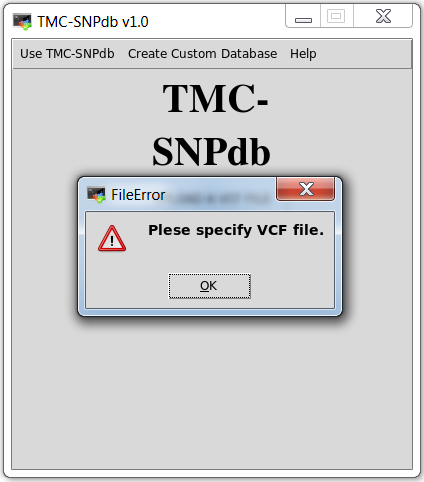

Supplement: Supplementary Data [file supp_baw104_suppl_data.zip › Supplementary File 1.docx]
