# Supplementary material for: TMC-SNPdb: an Indian germline variant database derived from whole exome sequences
Source: Database (Oxford). 2016 Jul 9;2016:baw104. doi: 10.1093/database/baw104 (PMC4940432; doi:10.1093/database/baw104)
Supplement: Supplementary Data [file supp_baw104_suppl_data.zip › Supplementary Table 1.docx]

**Supplementary Table 1: Whole exome sequencing data statistics of samples included in TMC-SNPdb.**

| S.No | ADLAB_ID | Tissue type | Sample type | Library Kit | Number of reads | Mapped reads | Percent Mapped | Mean Coverage |
| --- | --- | --- | --- | --- | --- | --- | --- | --- |
| 1 | AD0697 | Cervical | Tissue | SureSelect v5 | 46358172 | 30871424 | 67 | 93 |
| 2 | AD0722 | Cervical | Tissue | SureSelect v5 | 37346018 | 28005048 | 74 | 85 |
| 3 | AD0703 | Cervical | Tissue | SureSelect v5 | 40668628 | 25570273 | 63 | 77 |
| 4 | AD0704 | Cervical | Tissue | SureSelect v5 | 38010492 | 24847592 | 65 | 75 |
| 5 | AD0711 | Cervical | Blood | SureSelect v5 | 42307838 | 29243577 | 69 | 88 |
| 6 | AD0719 | Cervical | Blood | SureSelect v5 | 67952764 | 46526964 | 68 | 141 |
| 7 | AD0700 | Cervical | Tissue | SureSelect v5 | 90430850 | 89418505 | 99 | 179 |
| 8 | AD0689 | Cervical | Tissue | SureSelect v5 | 90177536 | 89229356 | 99 | 178 |
| 9 | AD0693 | Cervical | Tissue | SureSelect v5 | 96782488 | 95824313 | 99 | 192 |
| 10 | AD0695 | Cervical | Tissue | SureSelect v5 | 54056398 | 53213454 | 98 | 106 |
| 11 | AD0707 | Cervical | Blood | SureSelect v5 | 82318198 | 81601296 | 99 | 163 |
| 12 | AD0709 | Cervical | Tissue | SureSelect v5 | 32992728 | 32733386 | 99 | 65 |
| 13 | AD0714 | Cervical | Blood | SureSelect v5 | 66391960 | 65873552 | 99 | 132 |
| 14 | AD0716 | Cervical | Blood | SureSelect v5 | 84342838 | 83428663 | 99 | 167 |
| 15 | AD0717 | Cervical | Blood | SureSelect v5 | 89481162 | 88588499 | 99 | 177 |
| 16 | AD0691 | Cervical | Tissue | SureSelect v5 | 87667854 | 86708681 | 99 | 173 |
| 17 | AD0698 | Cervical | Tissue | SureSelect v5 | 77698290 | 76869536 | 99 | 154 |
| 18 | AD0699 | Cervical | Tissue | SureSelect v5 | 57855550 | 57156699 | 99 | 114 |
| 19 | **AD0746 | Cervical | Blood | TruSeq v2 | 4229630 | 3728161 | 88 | 6 |
| 20 | **AD0792 | Cervical | Blood | TruSeq v2 | 2434825 | 1803554 | 74 | 3 |
| 21 | AD0793 | Cervical | Blood | TruSeq v2 | 49920373 | 49482640 | 99 | 57 |
| 22 | AD0794 | Cervical | Blood | TruSeq v2 | 44251517 | 43841324 | 99 | 51 |
| 23 | AD0795 | Cervical | Blood | TruSeq v2 | 25106181 | 22167990 | 88 | 26 |
| 24 | AD0797 | Cervical | Blood | TruSeq v2 | 51917164 | 49171428 | 95 | 29 |
| 25 | AD0798 | Cervical | Blood | TruSeq v2 | 23676945 | 22211029 | 94 | 13 |
| 26 | AD0806 | Cervical | Blood | TruSeq v2 | 35855610 | 35015679 | 98 | 56 |
| 27 | AD0764 | Cervical | Blood | TruSeq v2 | 37472890 | 36799409 | 98 | 59 |
| 28 | AD0762 | Cervical | Blood | TruSeq v2 | 31341238 | 30807228 | 98 | 50 |
| 29 | AD0752 | Gallbladder | Tissue | SureSelect v5 | 63858844 | 35885515 | 56 | 108 |
| 30 | AD0759 | Gallbladder | Tissue | SureSelect v5 | 43455624 | 27369663 | 63 | 83 |
| 31 | AD0754 | Gallbladder | Tissue | SureSelect v5 | 54998184 | 37809365 | 69 | 114 |
| 32 | AD0755 | Gallbladder | Tissue | SureSelect v5 | 66115458 | 47741170 | 72 | 144 |
| 33 | AD0788 | Gallbladder | Tissue | SureSelect v5 | 39594984 | 23065796 | 58 | 70 |
| 34 | AD0757 | Gallbladder | Tissue | SureSelect v5 | 51254660 | 34364520 | 67 | 104 |
| 35 | AD0437 | Gallbladder | Tissue | SureSelect v5 | 44676668 | 29362140 | 66 | 89 |
| 36 | AD0439 | Gallbladder | Tissue | SureSelect v5 | 49994928 | 29952529 | 59 | 90 |
| 37 | AD0746 | Gallbladder | Tissue | SureSelect v5 | 37563776 | 21449942 | 57 | 65 |
| 38 | AD0761 | Gallbladder | Tissue | SureSelect v5 | 43581276 | 28263519 | 64 | 85 |
| 39 | AD0763 | Gallbladder | Tissue | SureSelect v5 | 37563776 | 21449942 | 57 | 65 |
| 40 | AD0725 | tongue | Tissue | TruSeq v3 | 33260084 | 32260230 | 97 | 52 |
| 41 | AD0775 | tongue | Tissue | TruSeq v3 | 28727389 | 27914222 | 97 | 45 |
| 42 | AD0744 | tongue | Tissue | NimbleGen | 66015439 | 63720122 | 97 | 101 |
| 43 | AD0771 | tongue | Tissue | TruSeq v3 | 7380086 | 5736294 | 78 | 9 |
| 44 | AD0782 | tongue | Tissue | NimbleGen | 74188836 | 72453644 | 98 | 115 |
| 45 | AD0777 | tongue | Tissue | NimbleGen | 46637332 | 45169670 | 97 | 72 |
| 46 | AD0783 | tongue | Tissue | NimbleGen | 65866424 | 64340950 | 98 | 102 |
| 47 | AD0778 | tongue | Tissue | NimbleGen | 50733602 | 49388815 | 97 | 78 |
| 48 | AD0779 | tongue | Tissue | NimbleGen | 34344688 | 33508795 | 98 | 53 |
| 49 | AD0780 | tongue | Tissue | NimbleGen | 70964617 | 69346504 | 98 | 110 |
| 50 | AD0784 | tongue | Tissue | NimbleGen | 82425599 | 80259390 | 97 | 127 |
| 51 | AD0731 | tongue | Tissue | NimbleGen | 69614600 | 67126889 | 96 | 107 |
| 52 | AD0786 | tongue | Tissue | NimbleGen | 75480562 | 73581363 | 97 | 117 |
| 53 | AD0787 | tongue | Tissue | NimbleGen | 67960054 | 66555223 | 98 | 106 |
| 54 | AD0781 | tongue | Tissue | NimbleGen | 80188961 | 78116302 | 97 | 124 |
| 55 | AD0733 | tongue | Tissue | NimbleGen | 64055460 | 61981799 | 97 | 98 |
| 56 | AD0734 | tongue | Tissue | NimbleGen | 75054746 | 72899349 | 97 | 116 |
| 57 | AD0720 | tongue | Tissue | TruSeq v3 | 31016587 | 13746645 | 44 | 22 |
| 58 | *****AD0721 | tongue | Tissue | TruSeq v3 | 7161446 | 6259124 | 87 | 10 |
| 59 | *****AD0772 | tongue | Tissue | TruSeq v3 | 12684423 | 11584458 | 91 | 19 |
| 60 | AD0773 | tongue | Tissue | TruSeq v3 | 76225980 | 66292936 | 87 | 107 |
| 61 | AD0774 | tongue | Tissue | TruSeq v3 | 32504474 | 30146066 | 93 | 49 |
| 62 | AD0789 | tongue | Tissue | NimbleGen | 34939297 | 34104607 | 98 | 54 |

In the ADLAB_ID column ** asterisk denotes sample with low coverage due to high duplication rate, * due to low data yield by sequencing using ` Illumina GAIIx in separate run.
