# Supplementary figures and images for: TMC-SNPdb: an Indian germline variant database derived from whole exome sequences
Source: Database (Oxford). 2016 Jul 9;2016:baw104. doi: 10.1093/database/baw104 (PMC4940432; doi:10.1093/database/baw104)

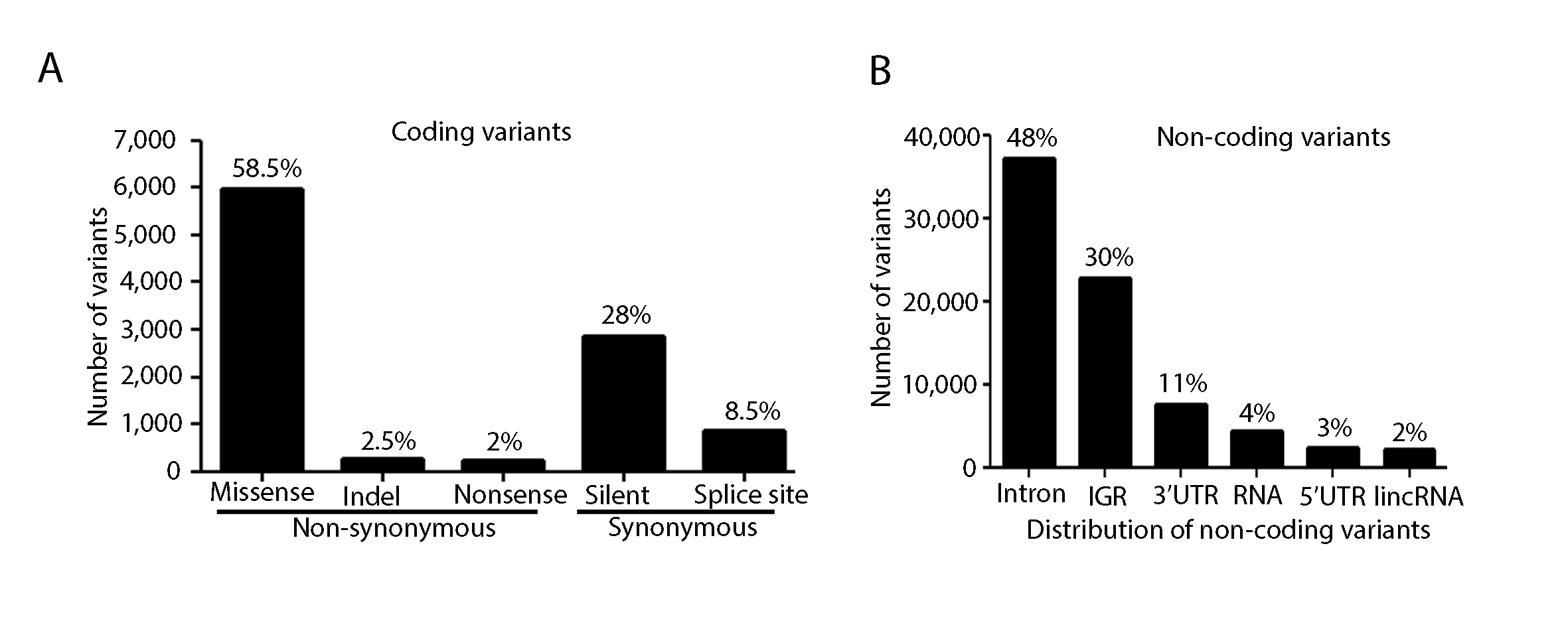

Supplement: Supplementary Data [file supp_baw104_suppl_data.zip › Supplementary Fig 1.tif]

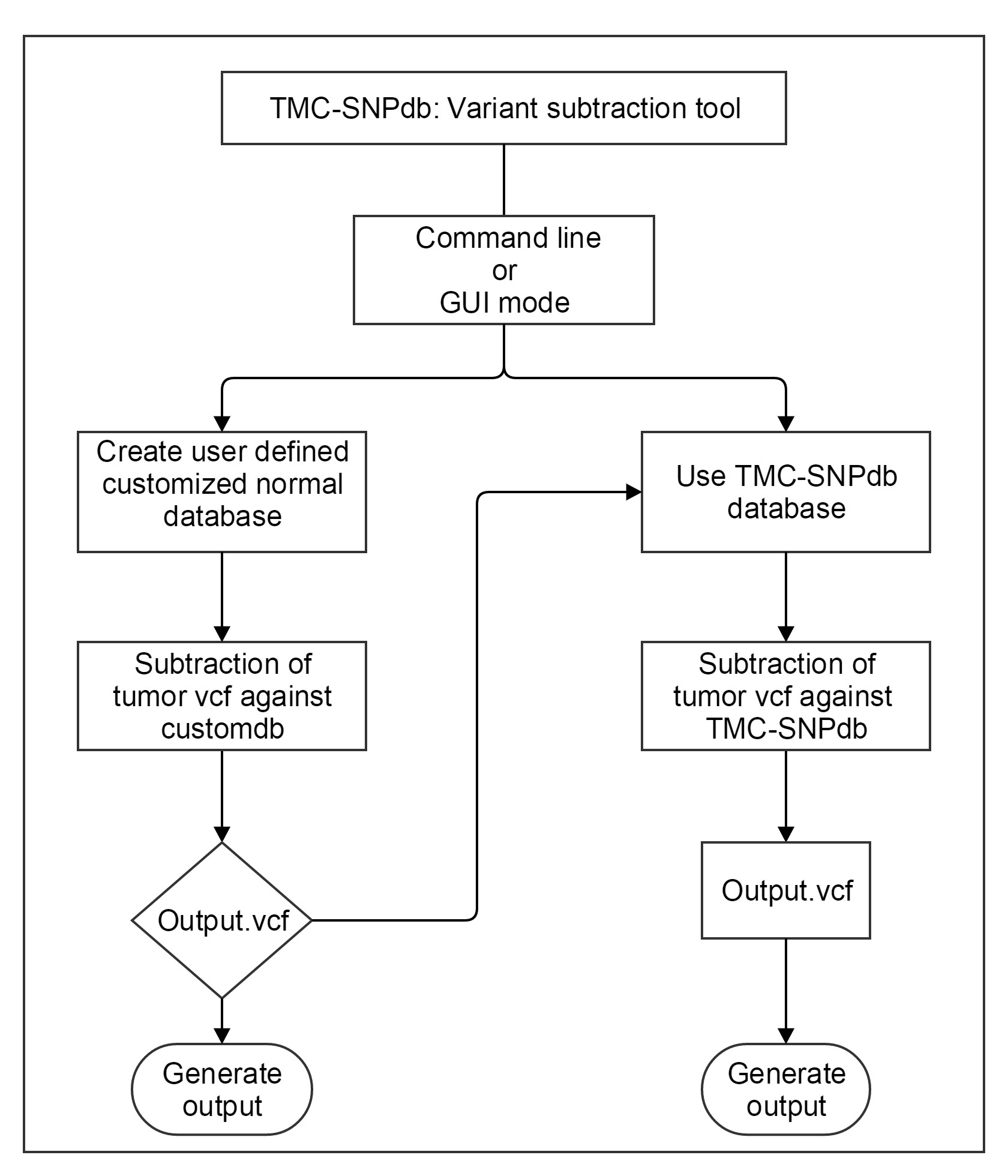

Supplement: Supplementary Data [file supp_baw104_suppl_data.zip › Supplementary Fig 2.jpg]

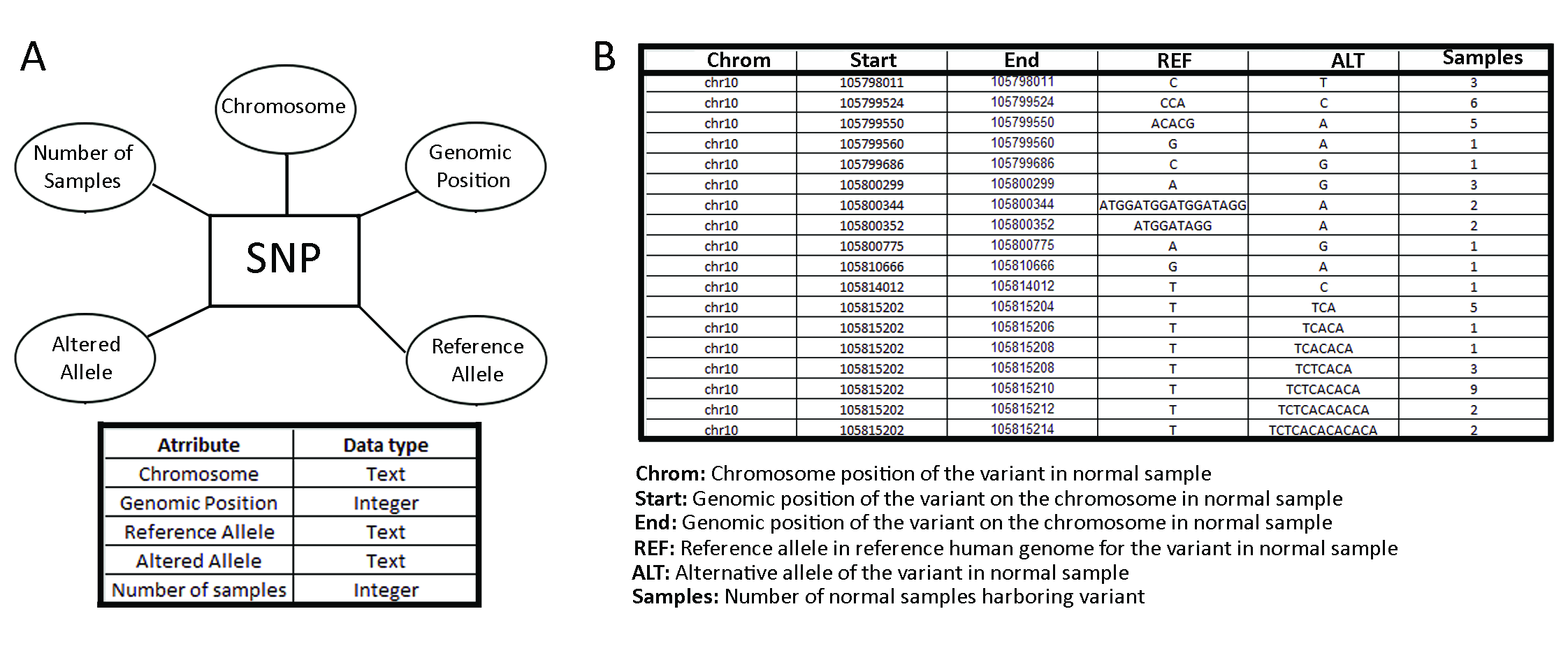

Supplement: Supplementary Data [file supp_baw104_suppl_data.zip › Supplementary Figure 3.tif]
